# Increased TRIM31 gene expression is positively correlated with SARS-CoV-2 associated genes TMPRSS2 and TMPRSS4 in gastrointestinal cancers

**DOI:** 10.1038/s41598-022-15911-2

**Published:** 2022-08-15

**Authors:** Mehmet Arda Temena, Ahmet Acar

**Affiliations:** Department of Biological Sciences, Middle East Technical University, Universiteler Mah. Dumlupınar Bulvarı 1, 06800 Çankaya, Ankara Turkey

**Keywords:** Computational biology and bioinformatics, Data processing

## Abstract

Besides typical respiratory symptoms, COVID-19 patients also have gastrointestinal symptoms. Studies focusing on the gastrointestinal tumors derived from gastrointestinal tissues have raised a question whether these tumors might express higher levels of SARS-CoV-2 associated genes and therefore patients diagnosed with GI cancers may be more susceptible to the infection. In this study, we have analyzed the expression of SARS-CoV-2 associated genes and their co-expressions in gastrointestinal solid tumors, cancer cell lines and patient-derived organoids relative to their normal counterparts. Moreover, we have found increased co-expression of TMPRSS2-TMPRSS4 in gastrointestinal cancers suggesting that SARS-CoV-2 viral infection known to be mediated by this protease pair might facilitate the effects of viral infection in GI cancer patients. Further, our findings also demonstrate that TRIM31 expression is upregulated in gastrointestinal tumors, while the inhibition of TRIM31 significantly altered viral replication and viral processes associated with cellular pathways in gastrointestinal cancer samples. Taken together, these findings indicate that in addition to the co-expression of TMPRSS2-TMPRSS4 protease pair in GI cancers, TRIM31 expression is positively correlated with this pair and TRIM31 may play a role in providing an increased susceptibility in GI cancer patients to be infected with SARS-CoV-2 virus.

## Introduction

Severe acute respiratory syndrome coronavirus 2 (SARS-CoV-2) which can cause asymptomatic, mild to moderate and severe respiratory coronavirus disease 2019 (COVID-19) infections emerged in Wuhan (China) in December 2019^1,2^. SARS-CoV-2 has infected approximately 400 million people to date (by February 2022) and caused more than 5,7 million deaths^3^. Right after its emergence, the COVID-19 pandemic has started to transform the lives of people and profoundly altered medicinal approaches in the treatment of patients. The lack of widespread access to effective vaccines worldwide and the emergence of new variants have raised new concerns regarding the transmission of the virus^4,5^. Furthermore, different clinical responses to viral or other infections in some of the patients are known to be regulated by genetic factors or interindividual variability^6^. In terms of the transmission pathways of the virus, the evasion from natural immunity and the high infectivity of new variants are the main causes for the continuous and rapid spread of SARS-CoV-2. In order to stop the spread of the virus, it is essential to uncover the underlying molecular mechanisms of immune evasion and infectivity^7–10^.

Direct fusion of the cell membrane with the viral envelope or membrane fusion within the endosomes following the endocytosis allows the virus to enter the cell^11^. SARS-CoV-2 binds to angiotensin-converting enzyme 2 (ACE2), which is widely expressed in many tissues in the human body^12,13^. Besides this receptor, other host receptor proteins facilitating virus entry into cells have been reported^11^. The spike glycoprotein that is responsible for the attachment of the virus to the host cell receptor, known as S protein, consists of one signal peptide, receptor-binding domain (RBD), subdomains in one subunit (S1) and heptad repeats with membrane-fusion subunit (S2)^14,15^. After RBD-receptor interaction, the S protein undergoes proteolysis which is catalyzed through interaction of host proteins/proteases like furin; paired basic amino acid cleaving enzyme FURIN, transmembrane serine protease 2 (TMPRSS2) and cathepsin B/L (CTSB/L)^16^. Apart from these, there are also co-receptors that can increase viral infectivity and have a significant role to facilitate binding characteristics in cells^17^. For example, NRP1 from the neuropilin (NRPs) family, which are highly conserved, and single-transmembrane proteins can act as a co-receptor for various molecules to facilitate the entry of SARS-CoV-2 into host cells^18–20^. Although SR-B1 (Scavenger Receptor Class B Member 1; SCARB1) cannot directly bind to the S protein, the SCARB1 expression facilitates SARS-CoV-2 entry into cells when co-expressed with ACE2 through utilizing cholesterol recognition motifs on the subunit S1^17,21^. Among other proteins, serine proteases are also involved in the infection process by interacting with the spike protein of the virus, one of which is TMPRSS4. Recent findings suggest that TMPRSS4 may play an important role for the coronavirus infection^22,23^.

Even though it is widely reported that COVID-19 affects the respiratory tract, associated with alveolar damage, extrapulmonary symptoms have also been observed^24^. Thrombotic diseases, dysfunction and rhythm disorders in the heart muscle, acute coronary syndromes, serious kidney damage, gastrointestinal symptoms, hepatocellular complications, ocular, dermatological and neurologic symptoms are among the other symptoms commonly seen in COVID-19 patients^24–26^. Due to high expression of ACE2 in extrapulmonary tissues, there is a possibility that SARS-CoV-2 can infect these tissues. As such, endothelial damage, and thrombo-inflammation in extrapulmonary tissues of COVID-19 patients may involve ACE2-related pathways such as TMPRSS2 alongside with the dysregulation of immune response^24,27,28^.

Although the symptoms caused by the virus are mostly seen in the respiratory tract, approximately 15% of the patients show gastrointestinal symptoms^29^. In addition to the high expression of ACE2 in intestinal glandular cells, elevated expression of viral nucleocapsid proteins in epithelial cells of gastric, duodenal and rectal tissues and glandular enterocytes can cause susceptibility of coronavirus infection in gastrointestinal tissues^30,31^. On the one hand, high expression of FURIN and TMPRSS2 in small intestinal epithelial cells mediates increased susceptibility to coronavirus infection in gastrointestinal tissues^32^. On the other hand, epithelial cells in the gut (mainly absorptive enterocytes of the colon and ileum) and esophagus also highly express ACE2 receptor^33,34^. Moreover, glandular cells of both the duodenum and the stomach have been reported to express ACE2; thus, SARS-CoV-2 can infect intestinal epithelial cells through ACE2 receptor^33^. Additionally, ACE2 receptors in the gastrointestinal tract play a crucial regulatory role in homeostasis, gut microbiome, and innate immunity. As a result, binding SARS-CoV-2 to these receptors and activating other epithelial surface proteins that can facilitate the entry of the virus may be one of the reasons for coronavirus infection in gastrointestinal tissues^35^. Conversely, live virus and RNA material that have been detected in the stools of COVID-19 patients were reported play a role in SARS-CoV-2 entry into cells starting from the apical surface and activating epithelial cell fusion in the intestine^36–38^. In particular, TMPRSS2 and TMPRSS4 serine proteases work together, accelerating S protein cleavage and thus increasing membrane fusion^22,39^.

It has also been observed that the intrinsic variability of receptors in cells and different tissue factors such as structural and genetic modifications on these proteins can affect a patient's susceptibility to COVID-19^40^. In one of the first reports of COVID-19 among cancer patients in China, it was reported that they experienced a serious risk of COVID-19, with other complications in addition to respiratory distress, compared to patients without cancer^41^. Since most of the cancer patients suffer from post treatment side effects including impaired immune system, these patients are possibly more susceptible to COVID-19^42,43^. A recent study that investigated clinical manifestations of COVID-19, reported that cancer patients had a 3.5-fold increase in the risk of morbidity and an increased risk of severe infection compared to non-cancer patients^41^. In addition to this, according to one comprehensive study, authors reported that cancer patients who have encountered severe acute respiratory syndrome virus (SARS or SARS-CoV-1), Middle East Respiratory Syndrome (MERS) virus, and SARS-CoV-2 have a higher risk of severe complications and death^44^. In general, patients with cancer may be much more vulnerable to SARS-CoV-2 infection^45^. However, important clinical questions remain unanswered is that optimal treatment and prevention strategies for cancer patients exposed to or infected with SARS-CoV-2^46^. In addition to COVID-19 risk in general cancer patient population, there have been studies demonstrating the risk of COVID-19 infection in GI cancer patients. Aznab et al., investigated COVID-19 prevalence in 279 cancer patients and reported that 72 CRC patients had COVID-19 infection while 11 patients with lung cancer, 5 with brain cancer and 12 with ovarian cancer had COVID-19 infection^47^. Another study performed a meta-analysis including 6 different studies to evaluate the prevalence of COVID-19 infection in cancer patients^48^. This study reported that prevalence of COVID-19 infection in a total of 205 cancer patients including GI cancer types (CRC, Esophagus and Pancreatic cancers) and they found that prevalence of COVID-19 infection in CRC patients was 20.5%, in Esophagus cancer patients was 7.6%, in Pancreatic cancer patients was 6.1%, and in lung cancer patients was 24.7%. Another metanalysis study showed that the prevalence of COVID-19 infection in CRC patients was 45.1% by comparing 6 different studies composed of 204 different cancer patients^49^. Despite all these studies show high infection risk of COVID-19 for GI cancer patients, these findings should be approached with caution due to following factors. Estimating COVID-19 infection risk in cancer patients requires more patients involved in larger studies. In addition, such studies need to take into consideration whole population beyond cancer patients with COVID-19 infection. Moreover, ethnicity bias may play role as these studies mainly rely on data obtained from few developed countries.

In this study, we performed a systematic gene expression analysis for SARS-CoV-2 genes in gastrointestinal tumor samples using publicly available datasets of solid tumors, cell lines and patient-derived organoids relative to their normal counterparts. In addition to individual gene expression changes in majority of GI cancers for *TMPRSS2*, *TMPRSS4*, *ACE2*, *CTSL, NRP1, FURIN, and SCARB1*, we assessed co-expression of highly expressed genes. We found that co-expressed *TMPRSS2* and *TMPRSS4* gene pair is positively correlated in GI samples. Moreover, our findings demonstrate that *TRIM31* is positively correlated with *TMPRSS2*-*TMPRSS4* genes in GI samples suggesting a possibility of *TRIM31* acting alongside with *TMPRSS2*-*TMPRSS4* protease pair in SARS-CoV-2 viral entry and promoting viral infection in GI cells. Finally, *TRIM31* knockdown data obtained from colorectal cancer cell lines resulted in changes in cellular pathways involved in viral replication and viral processes. This supports our hypothesis whereby *TRIM31* may be linked to processes that mediate SARS-CoV-2 infection in GI cancers.

## Results

**Figure 1:**
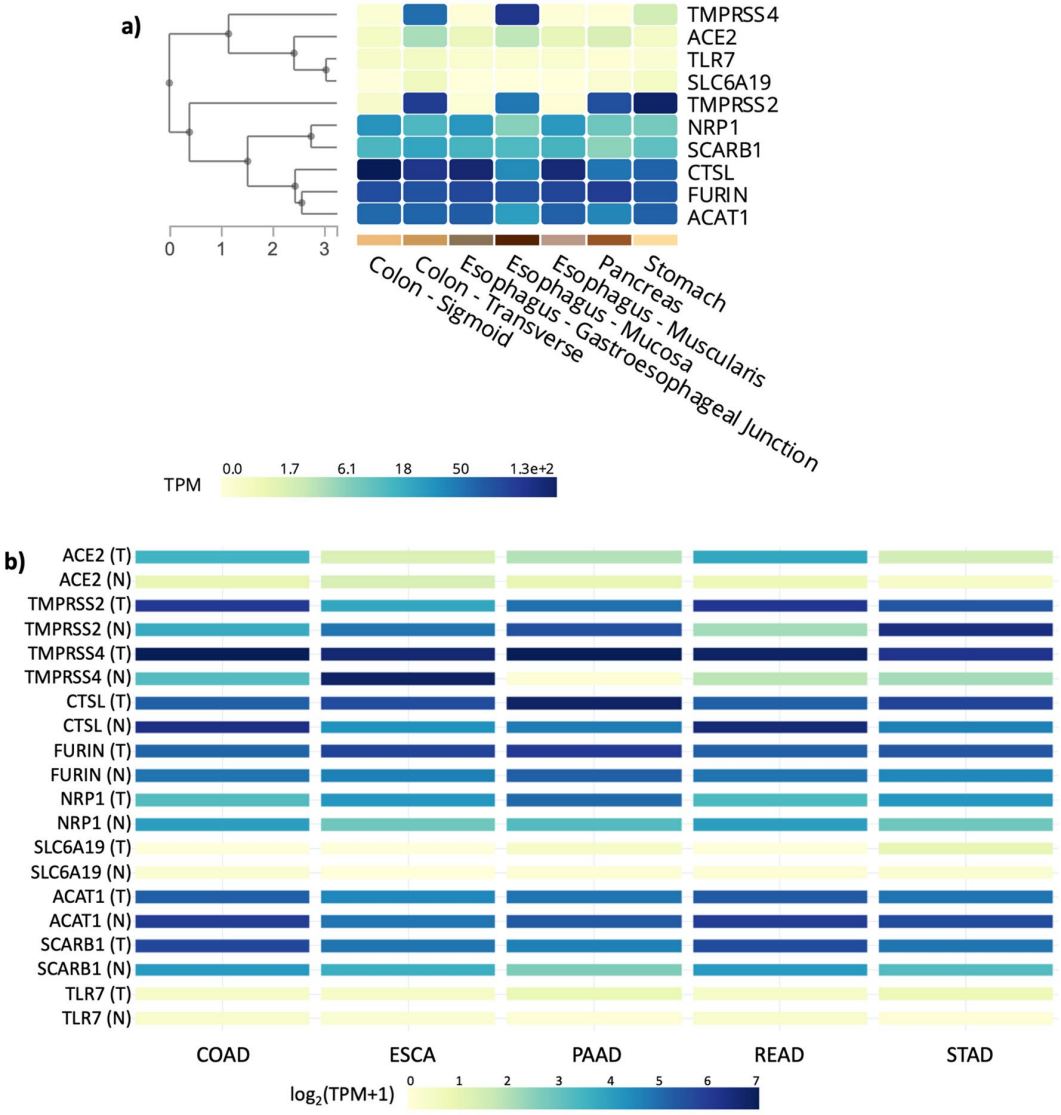
Heatmap of RNA-sequencing-based gene expression from the SARS-CoV-2-associated genes generated using GTEx portal for a multi-gene query in seven gastrointestinal tissues. (**a**) Comparison of the genes in different gastrointestinal normal samples. (**b**) TCGA tumor-normal samples and GTEx samples together (*T* tumor, *N* normal, *TPM* Transcripts per kilobase million-expresses RNA-sequencing reads normalized for the length of gene and sequencing depth).

Gene Set Enrichment Analysis-Molecular Signatures DataBase (GSEA-MSigDB) was utilized to obtain SARS-CoV-2 infection canonical pathway genes. The resulting genes are documented in Table 1. These genes were reported as responsible for the attachment of SARS-CoV-2 and its entry into cells. Initially we performed comparative gene expression analysis between normal gastrointestinal (GI) tissue samples. The analysis of gene expression changes between normal tissue samples revealed that *CTSL* expression in colon and esophagus mucosa, FURIN expression in pancreas, and *TMPRSS2* expression in stomach was found as the highest among other genes (Fig. 1a). We next investigated the gene expression changes in GI tumor samples in comparison to their normal derivatives and observed significant changes in the expression of SARS-CoV-2-associated genes in various GI tumors (Fig. 1b). When tumor tissue samples were analyzed in comparison to normal samples, colon adenocarcinoma (COAD) and rectal adenocarcinoma (READ) samples exhibited significantly elevated expression of *ACE2, SCARB1, TMPRSS2* and *TMPRSS4* genes while pancreatic adenocarcinoma (PAAD) and stomach adenocarcinoma (STAD) samples demonstrated increased expression of *ACE2, CTSL, NRP1*, *SCARB1* and *TMPRSS4* genes relative to normal samples (Fig. 1b). Further, *CTSL, FURIN, NRP1* and *SCARB1* gene expression levels were significantly higher in esophageal carcinoma (ESCA) samples in comparison to their normal matches (Fig. 1b). Differential gene expression results for each gene were given in supplementary figures in the following order: *ACE2* (Supplementary Figure S1a), *TMPRSS2* (Supplementary Figure S1b), *TMPRSS4* (Supplementary Figure S1c), *CTSL* (Supplementary Figure S1d), *FURIN* (Supplementary Figure S1e), *NRP1* (Supplementary Figure S1f), *SLC6A19* (Supplementary Figure S1g), *ACAT1* (Supplementary Figure S1h), *SCARB1* (Supplementary Figure S1i), *TLR7* (Supplementary Figure S1j).

**Table 1:** Gene set that was found in SARS-CoV-2 entry into host cells.

Gene symbol	Gene description
*CTSL*	Cathepsin L
*SLC6A19*	Solute carrier family 6 member 19
*ACAT1*	Acetyl-CoA acetyltransferase 1
*FURIN*	Paired basic amino acid cleaving enzyme
*TLR7*	Toll like receptor 7
*TMPRSS4*	Transmembrane serine protease 4
*ACE2*	Angiotensin I converting enzyme 2
*TMPRSS2*	Transmembrane serine protease 2
*NRP1*	neuropilin 1
*SCARB1*	Scavenger receptor class B member 1

**Figure 2:**
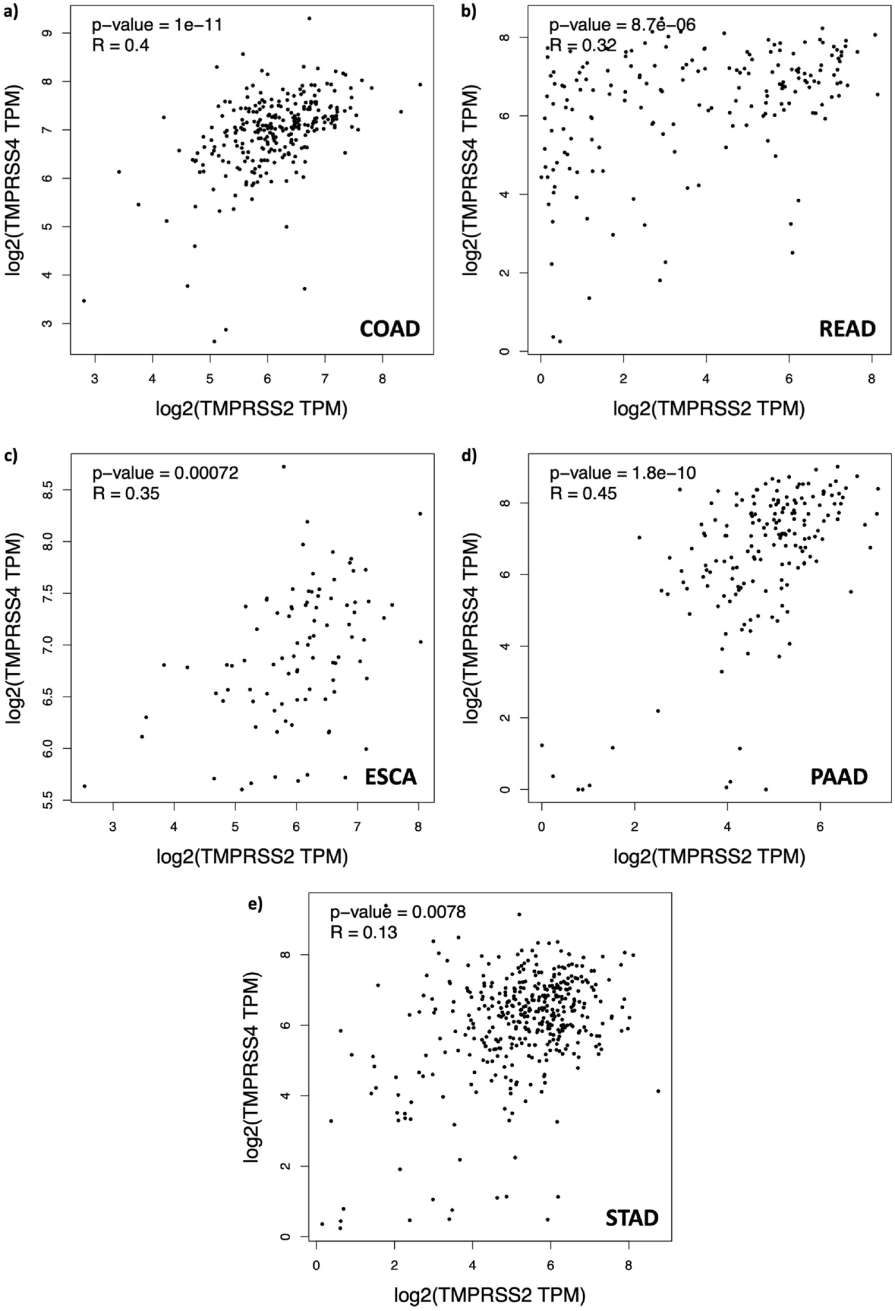
Correlation analysis results of *TMPRSS2* and *TMPRSS4* genes in gastrointestinal cancer tissues. Relationship of *TMPRSS2* and *TMPRSS4* with respect to their expression values shows moderate positive correlation (R-value > 0.3) in COAD, READ, ESCA and PAAD (**a**–**d**) and weak positive correlation in STAD samples (**e**).

After highly expressed genes were determined in GI solid tumor samples, a pairwise correlation analysis was performed to determine the relationship between these genes. Given some of the genes are co-expressed in tumor samples (Supplementary Figure S2), we reasoned that it may be possible to examine gene expression dependencies through their correlation analysis. After a pairwise correlation analysis of TCGA samples, *ACE2-SCARB1*, *TMPRSS2-SLC6A19* and *TMPRSS2-TMPRSS4* had a moderate positive correlation in COAD samples (Supplementary Figure S3a, p-values < 0.05 for all three; and R-values = 0.49, 0.32 and 0.4, respectively). For READ samples, in addition to positive correlation between *ACE2-SCARB1*, *TMPRSS2-SLC6A1*9 and *TMPRSS2-TMPRSS4*, a moderate positive correlation between *SCARB1-ACAT1* genes were also observed (Supplementary Figure S3b, p-values < 0.05 for all four; and R-values = 0.42, 0.5, 0.35 and 0.48, respectively). Moreover, we noted a moderate positive correlation between expression of *ACE2-TMPRSS2*, *TMPRSS2-TMPRSS4*, *TMPRSS2-SLC6A19*, *ACE2-SCARB1*, *CTSL-NRP1*, *NRP1-TLR7* and *SCARB1-ACAT1* in ESCA samples (Supplementary Figure S3c, p-values < 0.05 for all seven; and R-values = 0.36, 0.32, 0.38, 0.3, 0.39, 0.35 and 0.38, respectively). For PAAD samples, we observed a moderate positive correlation between *TMPRSS2-TMPRSS4, CTSL-TLR7, CTSL-NRP1* and *NRP1-TLR7* (Supplementary Figure S3d, p-values < 0.05 for all seven; and R-values = 0.45, 0.31, 0.39 and 0.62, respectively). Lastly, only *CTSL-NRP1, CTSL-TLR7* and *NRP1-TLR7* genes showed a moderate positive correlation in STAD samples (Supplementary Figure S3e, p-values < 0.05 for all three; and R-values = 0.51, 0.39, and 0.46, respectively). Importantly, all gastrointestinal tumor samples showed a positive correlation between *TMPRSS2* and *TMPRSS4* genes (Fig. 2). All correlation results between gene pairs in GI tumor types are presented in a supplementary file (Supplementary Figure S3). Taken together, these findings demonstrate that there is a positive correlation between the expression of a list of gene pairs specific to GI tumor types. Among these, *TMPRSS2* and *TMPRSS4* was commonly observed in all gastrointestinal tumor types.

**Figure 3:**
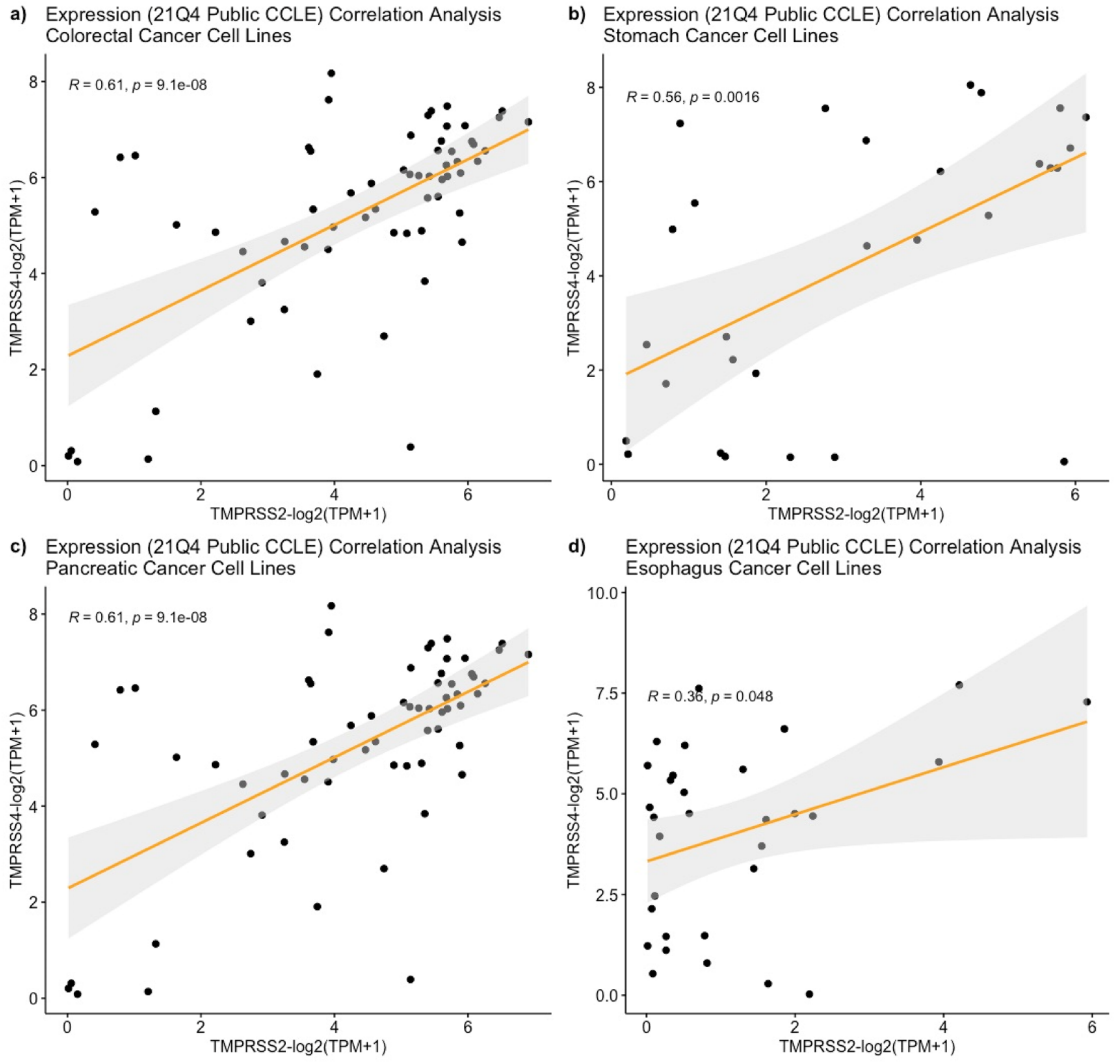
Positive correlation between *TMPRSS2* and *TMPRSS4* in gastrointestinal cancer cell lines from CCLE database. For colorectal (**a**), stomach (**b**) and pancreatic (**c**) cancer cell lines, R-value > 0.5 and for esophagus cancer cell lines (**d**) R-value > 0.3 while p-values for all of them < 0.05.

Following a pairwise gene expression correlation analysis using GI tumor samples, the same analysis was performed using the CCLE database. By doing this, we aimed at determining whether there is a correlation between the SARS-CoV-2-associated genes similar to our observations in GI tumor types. Initial analysis was performed to eliminate cell lines exhibiting zero gene expression. Following this, the cell lines that have at least log_2_(TPM + 1) > 0 gene expression were included into the analysis. As a result, 63 colorectal cancer, 32 esophagus cancer, 41 pancreatic cancer and 29 stomach cancer cell lines were used (Supplementary Table S1). When we performed gene expression correlation analysis for *TMPRSS2* and *TMPRSS4* genes in GI cancer cell lines, we found a moderate and statistically significant positive correlation for this gene pair in colorectal and stomach cancer cell lines (Fig. 3a, b p-values < 0.05 and R-values 0.613 and 0.559, respectively). Similarly, we observed statistically significant positive correlation for the expression of *TMPRSS2* and *TMPRSS4* genes in pancreatic and esophagus cancer cell lines (Fig. 3c, d p-values < 0.05 and R-values 0.359 and 0.521, respectively). These observations indicate that the expression of *TMPRSS2* and *TMPRSS4* gene pair are positively correlated to GI cancer cell lines. This resembles our findings in the GI tumor samples.

As *TMPRSS2* and *TMPRSS4* gene expression is positively correlated in both GI tumors and cell lines, we initially investigated whether there is an additional number of genes which are commonly associated with *TMPRSS2* and *TMPRSS4* genes in GI cancer cell lines. To address this question, we performed a correlation analysis in several GI cancer cell lines (colorectal, stomach, pancreatic, esophagus cancer cell lines) to identify a positively correlated gene(s) with *TMPRSS2* and *TMPRSS4*. We observed *TMPRSS2* and *TMPRSS4* expression to be positively correlated with seven genes which are Solute Carrier Family 44 Member 4 (*SLC44A4*), Proline Rich 15 Like (*PRR15L*), Receptor-interacting serine/threonine-protein kinase 3 (*RIPK3*), SH3 Domain Binding Glutamate Rich Protein Like 2 (*SH3BGRL2*), Transmembrane Protein 45B (*TMEM45B*), Tight Junction Protein 3 (*TJP3*) and Tripartite Motif Containing 31 (*TRIM31*) (Supplementary Table S2).

**Figure 4:**
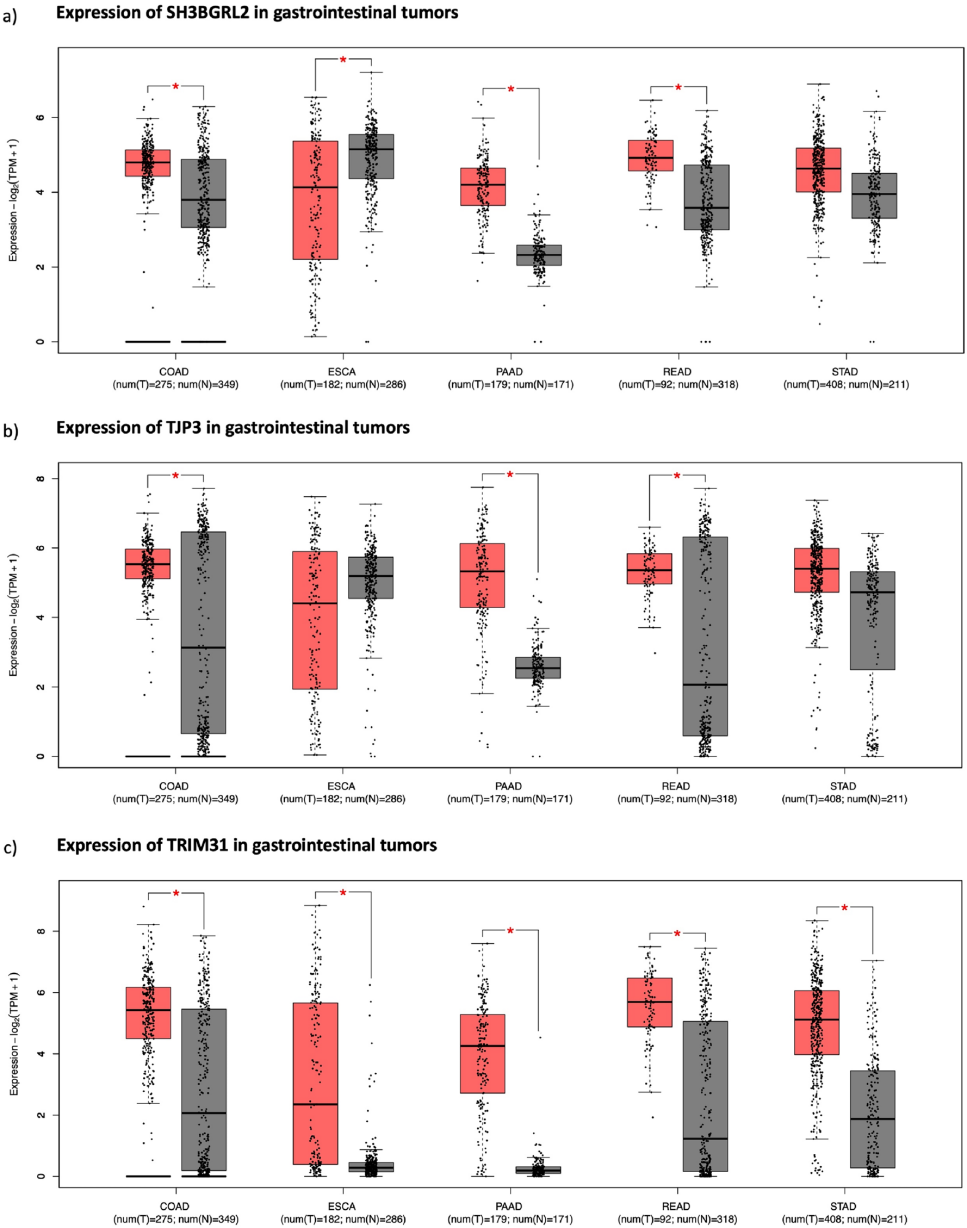
Cancer type-wise expression of eight genes (**a**; *SH3BGRL2*, **b**; *TJP3,* **c**; *TRIM31*) in TCGA gastrointestinal solid tumor samples. Red boxes are tumor samples while gray ones are normal and boxplots with ‘*’ sign shows statistically significant differential gene expression value, p-value < 0.05. Gene Expression Profiling Interactive Analysis: colon adenocarcinoma (COAD), rectal adenocarcinoma (READ), esophagus adenocarcinoma (ESCA), pancreas adenocarcinoma (PAAD), stomach adenocarcinoma (STAD).

We next investigated the differential gene expression of these seven genes in GI solid tumors in comparison to their matched normal tissue samples. All GI tumor samples except STAD showed significantly elevated expression of *SLC44A4* when compared to normal tissue samples (Supplementary Figure S4a). Moreover, *TMEM45B* expression was found to be significantly overexpressed in COAD, READ, STAD and PAAD except in ESCA samples (Supplementary Figure S4b). PRR15L showed highly increased expression in COAD and READ samples while *RIPK3 *was only significantly higher in PAAD samples relative to normal (Supplementary Figure S4c, Supplementary Figure S4d). In addition, no significant change or elevation in the expression of *SH3BGRL2* was found in STAD and ESCA samples while it was significantly overexpressed in COAD, READ and PAAD samples (Fig. 4a). Furthermore, TJP3 gene expression was found to be highly overexpressed in COAD, READ and PAAD samples in comparison to their matched normal samples (Fig. 4b). As a result, three genes, *SH3BGRL2, TJP3* and *TRIM31*, were positively correlated with *TMPRSS2* and *TMPRSS4* for all cancer cell lines and showed distinct differential expression profiles across GI solid tumors (Fig. 4). However, there was only one common gene that was both positively correlated with *TMPRSS2* and *TMPRSS4* in gastrointestinal cancer cell lines and significantly high in all gastrointestinal solid cancers, which was *TRIM31* (Fig. 4c). There was also significant positive correlation of *TRIM31* with *TMPRSS2* and *TMPRSS4* gene pair in all GI tumor samples (Supplementary Figure S5).

**Figure 5:**
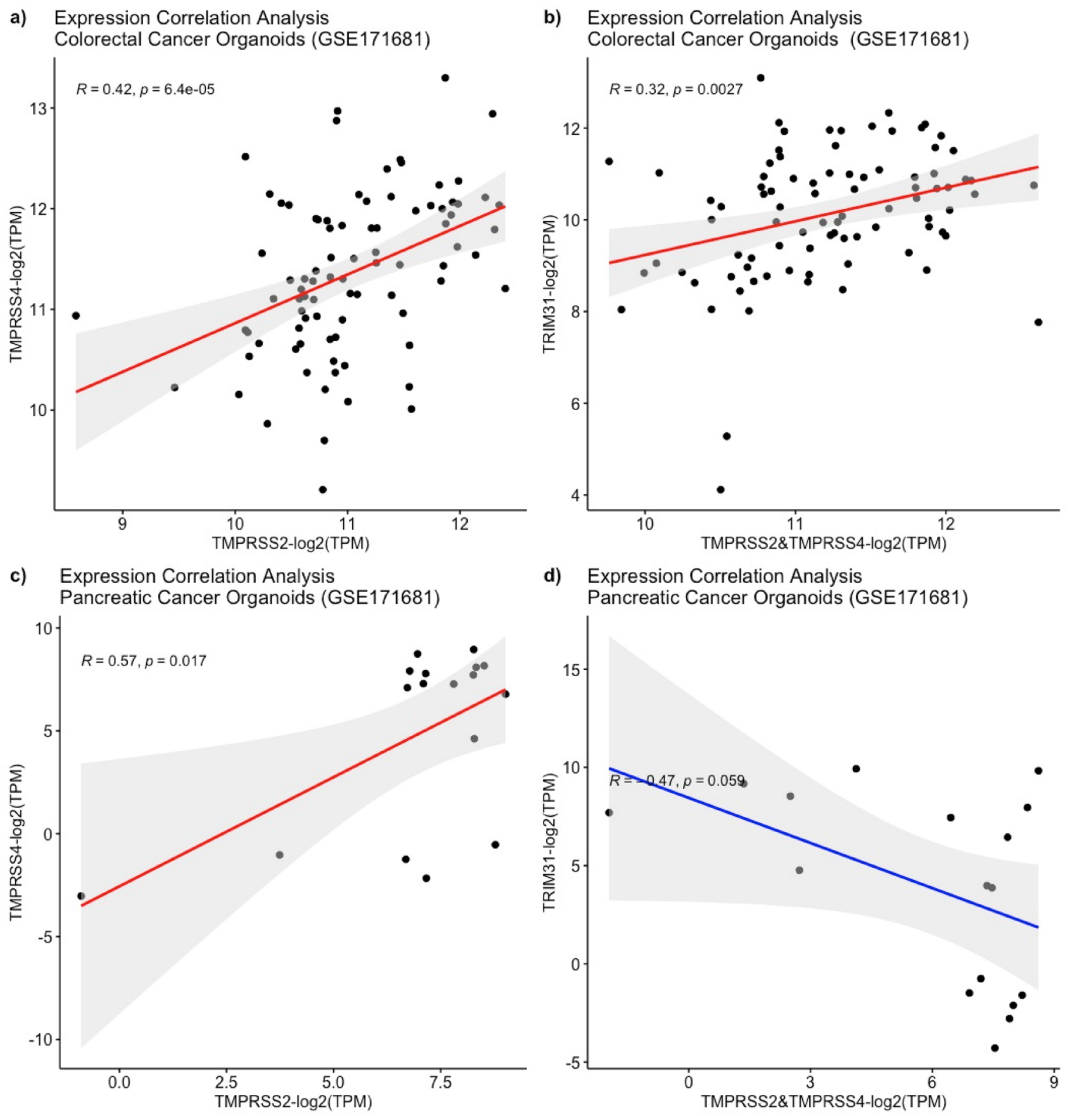
Correlation analysis of the *TMPRRS2, TMPRSS4* and *TRIM31* genes across patient derived organoids. Positive correlation between *TMPRSS2*&*TMPRSS4* and *TRIM31* was observed in colorectal cancer organoids (n = 87) (**a,b**). Only *TMPRSS2* shows a significant positive correlation with *TMPRSS4* (n = 17) but no significant correlation is observed between the serin protease pair and *TRIM31* in pancreatic cancer organoids (**c,d**).

After *TRIM31* was found to be upregulated and positively correlated with *TMPRSS2* and *TMPRSS4* genes in both cancer cell lines and solid tumor samples, we aimed to investigate the relationship of *TRIM31* with *TMPRSS2* and *TMPRSS4* using the clinically relevant model system Patient Derived Organoids (PDOs)^50,51^. RNA-seq expression counts data from 87 colorectal cancer PDOs with their matched tumor samples were retrieved from the Gene Expression Omnibus (GEO) database (GSE171681). Our pair-wise gene expression correlation analysis revealed that *TRIM31* gene expression in colorectal PDOs was positively correlated with TMPRSS2 and TMPRSS4 (Fig. 5a, b). Yet, there was no significant correlation observed between *TRIM31* to *TMPRSS2* and *TMPRSS4* in pancreatic cancer PDOs, but a positive correlation was still observed between *TMPRSS2* and *TMPRSS4* (Fig. 5c, d). Taken together, these observations indicate that *TRIM31* gene expression is positively correlated with *TMPRSS2* and *TMPRSS4* in GI cell lines, solid tumors and colorectal cancer PDOs.

**Figure 6:**
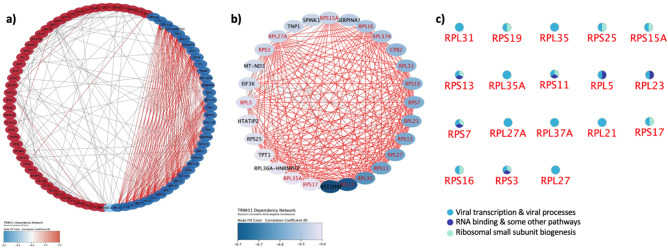
PPI network analysis for affected genes when *TRIM31* was silenced in 20 CRC cell lines using rnai. while upregulated genes with red circles exhibited weak co-expression score (less than 0.5), blue circles indicating downregulated genes showed co-expression score bigger than 0.5 (**a**). Blue circles exhibiting downregulated genes are highlighted with red coloured text exhibited 18 nodes indicating similar processes using gene ontology analysis (**b**). The 18 nodes indicating cellular processes that mostly affected by *TRIM31* silencing are presented by the names of these proteins and their cellular function such as viral transcription & viral processes, RNA binding, and ribosome small subunit biogenesis are listed (**c**). Majority of these cellular processes that are affected by *TRIM31* silencing in crc cell lines are related to viral transcription & viral processes.

This was followed by investigating the functional role of *TRIM31* and its relationship cellular pathways using a genome-wide loss of screen approach. RNAi results for *TRIM31* in colorectal cancer cell lines were obtained from the CCLE database utilizing DEMETER2^52–56^. This model integrates large-scale pooled RNAi screening data expression changes. The relationship between expression correlation values and *TRIM31* dependency was determined in 20 colorectal cancer cell lines (Supplementary Table S3). Using the dataset, we performed a pathway enrichment analysis to assess *TRIM31* knockdown effect in cellular processes in colorectal cancer cell lines. Initially we have determined upregulated and downregulated genes when *TRIM31* was silenced using the RNAi approach (Fig. 6a). This analysis showed that number of genes downregulated genes (colored with blue circles) had bigger co-expression scores than upregulated genes (colored with red circles). This was then followed by the identification of related pathways when silencing of *TRIM31* caused downregulation of genes marked with blue circles. This analysis demonstrated that the loss of TRIM31 affects viral transcription (GO:0019083) (p-Value = 2.36e−17) and viral processes (GO:0016032) (p-Value = 5.50e−08) with a false discovery rate; p-value < 0.05 (Fig. 6b). A list of downregulated genes interacting in 18 nodes are presented (Fig. 6c). Considering most of these genes are involved in viral transcription and viral processes, TRIM31 is likely to play a role in mediating viral transcription and viral processes in colorectal cancer. Due to the positive correlation of *TRIM31* with *TMPRSS2* and *TMPRSS4* genes, it may therefore cause higher susceptibility of viral infections, such as SARS-CoV-2, seen in cancer patients.

## Discussion

With the emergence and spread of SARS-CoV-2 since December 2019, global public health concerns have reached an unprecedented level. The infection of SARS-CoV-2 in gastrointestinal tissues was soon to be discussed by a number of researchers^26,30^. Tumors in relation to SARS-CoV-2 virus infection and their susceptibility to this viral infection are yet to be fully investigated. Given the high expression levels of ACE2 in the glandular cells of the intestine and the presence of viral nucleocapsid proteins in gastrointestinal epithelial cells and glandular enterocytes^57,58^, we aimed to understand whether SARS-CoV-2 entry-mediated pathways are involved in gastrointestinal tumors. Our results suggest that there is a positive correlation between gene expression levels of the members of SARS-CoV-2 entry-mediated pathways, namely TMPRSS2, TMPRSS4, and TRIM31, which are involved in the viral infection of gastrointestinal tumors, cell lines and patient-derived organoids in comparison to their matched normal counterparts.

The pathophysiology of SARS-CoV-2 infection in gastrointestinal tissues may depend on many factors^59–61^. Not only normal GI tissues but also GI tumors may easily be infected by pathogens due to their distinct mucosal membrane protein expression profiles. We therefore characterized the expression profiles of a number of genes involved in SARS-CoV-2 infection pathway and found higher *ACE2, TMPRSS2, TMPRSS4, SCARB1, CTSL* and *NRP1* expression in all GI tumor samples relative to their normal counterparts. Exceptions include *ACE2, TMPRSS2, TMPRSS4* genes in ESCA samples; *TMPRSS2* gene in STAD and PAAD samples; *CTSL* and *NRP1* genes in COAD and READ samples; and *FURIN* gene in COAD, READ, STAD and PAAD samples. Among the significantly high expression profiles of receptors and mediator proteins for SARS-CoV-2 entry into cells in GI tumors, we observed high co-expression of *TMPRSS2* and *TMPRSS4* in all GI tumors demonstrating that these mucosa specific serine proteases can facilitate the entry of the spike protein into the cell membrane. Importantly, higher co-expression of *TMPRSS2* and *TMPRSS4* relative to normal was also observed in a human gastrointestinal cancer cell line dataset and colorectal cancer organoids. The latter especially holds importance due to its clinically accepted role as a relevant model system.

Given that the co-expression analysis with *TMPRSS2-TMPRSS4* resulted in a positive correlation with *TRIM31* in all GI samples, we further investigated the functional role of TRIM31 in GI tumors. TRIM31 is a member of E3 ubiquitin ligase family and mediates various cellular functions in gastrointestinal tissues including antiviral immune response^62^ and tumorigenesis^63,64^. Despite the antiviral role of mitochondrial antiviral-signaling protein (MAVS), there have been various studies, recently shown in COVID-19 infection, demonstrating that MAVS complex can be limited to facilitate viral infection^65^. Amongst the role of inhibiting antiviral role of MAVS signalling, NEMO-like kinase was reported to induce the degradation of MAVS. In addition, PLK1 was reported to phosphorylate and destroy MAVS complex^66^. The dual role of MAVS complex can be context specific and especially in cancers, where immune dysregulation is commonly observed^67^, MAVS can be regulated differently than its antiviral function through the activation various phosphorylation events and hence limiting the antiviral function of MAVS complex. Another study also showed that antiviral immune response mediated by MAVS complex is opposed by SARS-CoV-2 nucleocapsid protein (NP), SARS-CoV-2-NP, to facilitate NP-mediated immune evasion in on colon cancer cell line CaCo-2^68^. Given the prominent consequences of COVID-19 resulting in immune deregulation and evasion^69^, antiviral immune response can be impeded and therefore viral infection can be promoted in cancer cells. Furthermore, another study particularly demonstrated that MAVS expression was downregulated in human colon cancer tissue samples^70^. Based on this study, we also checked MAVS expression in CRC samples and found that MAVS expression was significantly downregulated in CRC samples in comparison to normal samples (Supplementary Figure S6). Based on this finding and supporting studies^65,66^ opposing the antiviral function of MAVS signalling, we think that decreased MAVS expression can impede antiviral immune response. In the case of viral infection, attenuated levels of MAVS may result in promoting SARS-CoV-2 viral infection and consequently promoting the viral evasion. As the MAVS expression is downregulated, TRIM31 may not perform its function as it normally mediates antiviral immune response via its interaction with the MAVS complex. TRIM31 has been previously reported to play a role in activation of NF-κB signalling in colorectal cancer^71^. In addition, NF-κB signalling was shown to be activated by viral infection^72^ and play a role in COVID-19 pathogenicity^73–75^. In this process, TRIM31 may play a role in inducing the expression of NK-κB and therefore the activation of inflammation might contribute to the viral processes. To support the role of TRIM31 in regulation viral processes, in Fig. 6, we show that inhibition of TRIM31 in colon cancer cell lines results in downregulation of viral transcription/processes related cellular pathways.

In addition, TMPRSS4 alongside with TMPRSS2 mediate SARS-CoV-2 viral entry in gut epithelial cells^76^ and highly expressed in GI cancer types^77^. These two proteases facilitating viral entry are co-expressed with TRIM31 which can act as promoting viral transcription and viral processes related pathways. To support this notion, we have shown that MAVS normally interacting with TRIM31 to facilitate antiviral immune response is downregulated in CRC samples (Supplementary Figure S6) and therefore TRIM31 may not function as it is normally reported in antiviral immune response. Besides the expression of *TMPRSS2*, *TMPRSS4*, and *TRIM31* in GI cancers, we have analysed the expression of these genes in all cancers, and we have found that the expression of these genes is more upregulated in majority of GI cancers than individual other cancer types (Supplementary Figure S7, Supplementary Figure S8, Supplementary Figure S9). It is important to note that we cannot rule out the possibility that other cancer types might also have increased susceptibility to COVID-19 infection. For example, other cancer types such as Lung Adenocarcinoma (LUAD) or Liver Hepatocellular Carcinoma (LIHC) may also exhibit increased susceptibility to COVID-19 infection via similar mechanisms governed by TMPRSS2, TMPRSS4, and TRIM31, however this remains to be further investigated.

*TRIM31* RNAi expression profile obtained from 20 colorectal cancer cell lines using CCLE dataset provided evidence for *TRIM31* dependency in these cell lines. Interaction network analysis performed following the *TRIM31* knockdown in colorectal cancer cell lines highlighted a possible functional role of TRIM31 in viral replication and viral processes. This may therefore be important to develop strategies for targeting TRIM31 particularly in colorectal tumors. Studies showed that protease inhibitors such as against TMPRSS2, CTSL or other proteases may play a role in tackling SARS-CoV-2 viral entry into cells as these proteases are known to regulate viral infection^78–80^. For example, camostat-mesylate, a TMPRSS2 inhibitor, was found to be successful for the inhibition of viral entry into lung epithelial cells^27^. In the light of our findings where *TRIM31* is positively correlated with *TMPRSS2-TMPRSS4* in GI tumors and knockdown of *TRIM31* mediates viral replication and viral processes, developing inhibitors targeting TRIM31 may decrease viral infection efficiency of SARS-CoV-2 in colorectal cancer patients. Taken together, our findings demonstrate that *TMPRSS2-TMPRSS4-TRIM31* genes are positively correlated together in colorectal cancer which may facilitate SARS-CoV-2 entry into cells. Hence, targeting TRIM31 may play an important role in preventing SARS-CoV-2 viral infection in colorectal cancer patients.

## Methods

### Data acquisition

No datasets were generated during the current study. The publicly available TCGA datasets were directly used from the TCGA Data Portal at (https://www.cancer.gov/tcga) for gastrointestinal solid cancers through Gene Expression Profiling Interactive Analysis portal^81^. For normal tissue samples, GTEx database (https://www.gtexportal.org/home) was used to identify expression values of selected genes in normal samples corresponding gastrointestinal tumor regions. The Cancer Cell Line Encyclopedia (CCLE) database (https://depmap.org/portal/ccle) was used to retrieve processed large-scale NGS expression datasets of cancer cell lines^82–84^. Raw sequencing data of CCLE for the Ghandi M et al. 2019 publication is available through the Sequence Read Archive under accession number PRJNA523380^82^. Among whole encyclopaedia, 82 colorectal cancer, 38 esophageal cancer, 47 pancreatic cancer and 35 stomach (gastric) cancer cell lines were included in this study based on the CCLE database, the names of which are given in the supplementary file (Table S1). To further investigate the expression correlations, two independent studies and three sets of RNA-seq data from gastrointestinal tumour organoid studies (GSE171680, GSE171681^51^ and GSE139184^50^) were used to identify gene expression correlations. Transcriptomic datasets of patient derived organoid models for only colorectal and pancreatic cancer were used since there was no available gastric and esophagus tumour organoid data and processed transcriptomic datasets available at the National Centre for Biotechnology Information (NCBI) Gene Expression Omnibus (GEO) under accession numbers: GSE171680, GSE171681 and GSE139184 (https://www.ncbi.nlm.nih.gov/geo).

### Genes selection and differential expression

The receptor and transmembrane proteins/genes that facilitate the entry of SARS-CoV-2 into cells used in this study were selected through Molecular Signature Database of the Gene Set Enrichment (GSEA-msigdb) repository (https://www.gsea-msigdb.org/) query and the gene set in WP_SARSCOV2_AND_COVID19_PATHWAY was used^85^. For comparative and differential expression analyses, the gene list given in Table 1 was used for the analysis of TCGA datasets for solid tumor samples with their control matches and corresponding normal tissue samples in GTEx. Gene Expression Profiling Interactive Analysis portal associated with its python package was used to compare tumor samples with normal tissue^81^. The expression data were first log2(TPM + 1) transformed for differential analysis and the log_2_FC was identified as median difference between tumors and normal in this portal. Genes with higher |log_2_FC| values were considered differentially expressed genes. Gene expression values of GTEx samples (normal) were produced with RNA-SeQC v1.1.9 and built-in tool in their websites were used to generate a normal tissue heatmap^33^. Tissue-wise differential expression of one gene or a multi-gene signature in gastrointestinal cancer types (colon adenocarcinoma, COAD; esophagus carcinoma, ESCA; pancreatic adenocarcinoma, PAAD; rectal adenocarcinoma, READ; and stomach adenocarcinoma, STAD) was determined. For differential gene expression analysis for solid tumors of TCGA, log_2_(TPM) was used for logscale comparison and |log_2_FC| cut-off as 1 was applied. Moreover, some of the cell lines were excluded due to zero expression or no expression data available for corresponding genes.

### Gene set correlation analysis

A linear regression analysis (Pearson’s correlation) was performed to explore the relationship between differentially expressed genes for each dataset. To define the relationship between differentially expressed genes, continuous variables were analyzed using Pearson’s correlation analysis. For correlation analyses, the non-log scale was used for calculation and the log-scale axis was used for visualization in TCGA samples, in CCLE samples and in other datasets.

### Loss-of-function screens and network analysis

Dependency analysis for cancer cell lines was performed using RNAi dependency dataset (Achilles + DRIVE + Marcotte, DEMETER2). The resulting gene expression changes demonstrating only moderate or positive relationship were further investigated. Protein–protein interaction networks functional enrichment analysis was performed using STRING (https://string-db.org) by all types of interactions with highest confidence interaction score (0.900). Disconnected nodes were excluded from visualization.

### Statistical analysis

Pearson’s correlation was calculated and concluded as strong when R value was larger than 0.7, moderate when R value was between 0.3 and 0.7 and weak when R value was between 0 and 0.3. Data were transformed into log_2_(TPM) from raw TPM values for pancreatic cancer organoids data and filtered according to related genes by using denoted packages with RStudio. Boxplots were generated using one-way ANOVA via GEPIA2 portal using disease status (Tumor and Normal) as variables for calculating differential expression and p-value < 0.05 was considered as significant for all differential gene expressions and correlation analyses. General data handling, filtering, and plotting was performed using RStudio (http://www.rstudio.com) with installed packages such as dplyr, tidyverse and ggpubr^86–88^.

## Supplementary Information

Supplementary Information 1.

Supplementary Information 2.

## Data Availability

Previously published datasets including GTEx samples^89^, TCGA samples^90^, CCLE samples^82^, pancreatic cancer organoids^50^, and colorectal cancer organoids^51^ were used in this study. The RNA-Seq datasets of TCGA/GTEx data are available within the GEPIA2^81^ and weblinks to these datasets are as following https://portal.gdc.cancer.gov, and https://gtexportal.org/home, http://gepia2.cancer-pku.cn, respectively. The CCLE provides public access to genomic data, which are openly available at https://sites.broadinstitute.org/ccle/datasets. All processed transcriptomic datasets for tumor organoid samples are available at the National Centre for Biotechnology Information (NCBI) Gene Expression Omnibus (GEO) under accession numbers of GSE171680, GSE171681 and GSE139184 (https://www.ncbi.nlm.nih.gov/geo).
