# Supplementary material for: Increased TRIM31 gene expression is positively correlated with SARS-CoV-2 associated genes TMPRSS2 and TMPRSS4 in gastrointestinal cancers
Source: Sci Rep. 2022 Aug 15;12:11763. doi: 10.1038/s41598-022-15911-2 (PMC9378649; doi:10.1038/s41598-022-15911-2)
Supplement: Supplementary file 2 — Supplementary Information 2. [file 41598_2022_15911_MOESM2_ESM.docx]

**Increased TRIM31 gene expression is positively correlated with SARS-CoV-2 associated genes TMPRSS2 and TMPRSS4 in gastrointestinal cancers**

**Mehmet Arda Temena^1^, Ahmet Acar^1, *^**

**^1^** Department of Biological Sciences, Middle East Technical University, Universiteler Mah. Dumlupınar Bulvarı 1, 06800 Çankaya, Ankara, Turkey

*Corresponding author: acara@metu.edu.tr

**Key words:** SARS-CoV-2, COVID-19, gastrointestinal cancers, TRIM31, TMPRSS2, TMPRSS4

**Supplementary Figure S1. (a-j)** Differential expression results for each gene across all gastrointestinal types of cancer in Figure 1. Red boxes are tumor samples while gray ones are normal and boxplots with ‘*’ sign shows statistically significant different expression value, p-value < 0.05.

**Supplementary Figure S2.** Protein-protein co-expressions results for each gene in cells according to STRING. From Gene-Set Enrichment Analysis from WikiPathways through GSEAmsigdb, ACE2 is co-expressed with SLC6A19 and TMPRSS2; TMPRSS2 is co-expressed with ACE2 and TMPRSS4, TMPRSS4 is co-expressed with TMPRSS2 with respect to genes involved inSARS-CoV-2 and COVID-19 Pathway. Co-expression based on RNA expression patterns, and on protein co-regulation provided by ProteomeHD.

**Supplementary Figure S3. (a-e)** Expression correlation analysis of all genes in gastrointestinal solid tumours from TCGA.

**Supplementary Figure S4. (a-d)** Differential expression results for each gene across all gastrointestinal types of cancer. Red boxes are tumor samples while gray ones are normal and boxplots with ‘*’ sign shows statistically significant different expression value, p-value < 0.05.

**Supplementary Figure S5.** Expression correlation analysis of TRIM31 and TMPRSS2&TMPRSS4 in gastrointestinal tumors from TCGA samples.


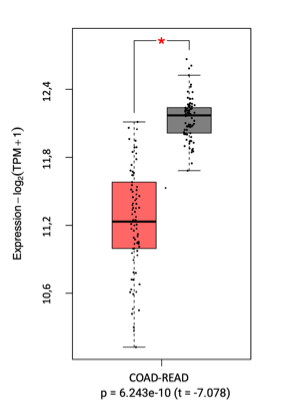

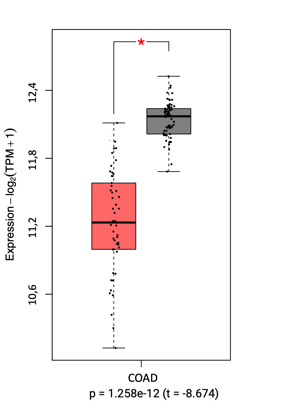


**Supplementary Figure S6.** MAVS gene expression changes in CRC and Colon Cancer samples from TCGA database. Red boxes are tumour samples while grey boxes are normal. Boxplots with “*” sign shows statistically significant differential gene expression value, p<0.05. CRC: COAD (colon adenocarcinoma) and READ (rectal adenocarcinoma), COAD.

**Supplementary Figure S7:** Differential gene expression results of TRIM31 gene across all cancer types. COAD (colon adenocarcinoma), ESCA (esophageal carcinioma), LIHC (liver hepatocellular carcinoma), PAAD (pancreatic adenocarcinoma), READ (rectal adenocarcinoma), STAD (stomach adenocarcinoma) indicated with bold showed statistically significant upregulation of TRIM31 expression in tumour samples in comparison to normal. Tumour samples are shown via red while normal samples via green colour. p-value < 0.05 was considered as significant.

**Supplementary Figure S8:** Differential gene expression results of TMPRSS2 gene across all cancer types. CESC (cervical squamous cell carcinoma), COAD (colon adenocarcinoma), KICH (kidney chromophobe), PRAD (prostate adenocarcinoma) READ (rectal adenocarcinoma), STAD (stomach adenocarcinoma), UCEC (uterine corpus endometrial carcinoma), and UCS (uterine carcinosarcoma) indicated with bold showed statistically significant upregulation of TMPRSS2 expression in tumour samples in comparison to normal. Tumour samples are shown via red while normal samples via green colour. p-value < 0.05 was considered as significant.

**Supplementary Figure S9:** Differential gene expression results of TMPRSS4 gene across all cancer types. CESC (cervical squamous cell carcinoma), COAD (colon adenocarcinoma), LUAD (lung adenocarcinoma) LUSC (lung squamous cell carcinoma), OV (ovarian cancer), PAAD (pancreatic adenocarcinoma), READ (rectal adenocarcinoma), STAD (stomach adenocarcinoma), THCA (thyroid cancer), UCEC (uterine corpus endometrial carcinoma), and UCS (uterine carcinosarcoma) indicated with bold showed statistically significant upregulation of TMPRSS4 expression in tumour samples in comparison to normal. Tumour samples are shown via red while normal samples via green colour. p-value < 0.05 was considered as significant.
